# An experimental study on the relation between friction force and real contact area

**DOI:** 10.1038/s41598-021-99909-2

**Published:** 2021-10-13

**Authors:** X. M. Liang, Y. Z. Xing, L. T. Li, W. K. Yuan, G. F. Wang

**Affiliations:** Department of Engineering Mechanics, SVL and MMML, Xi’an Jiaotong University, Xi’an, 710049 China

**Keywords:** Engineering, Mechanical engineering

## Abstract

Classical laws of friction suggest that friction force is proportional to the normal load and independent of the nominal contact area. As a great improvement in this subject, it is now widely accepted that friction force is proportional to the real contact area, and much work has been conducted based on this hypothesis. In present study, this hypothesis will be carefully revisited by measuring the friction force and real contact area in-site and real-time at both normal loading and unloading stages. Our experiments reveal that the linear relation always holds between friction force and normal load. However, for the relation between friction force and real contact area, the linearity holds only at the loading stage while fails at the unloading stage. This study may improve our understanding of the origin of friction.

## Introduction

Approximate 20 percent of the global energy consumption is attributed to harmful friction^1^, and it is of practical significance to improve the fundamental understanding of friction. Back in 1493, Leonardo da Vinci observed that *the friction force between two solids is proportional to the applied normal load* and independent of the nominal contact area^2^. The proportionality was hereby defined as the friction coefficient. The friction coefficient might be altered by the velocity of relative motion^3^, the topography of the contact interface^4^, the thickness of the lubrication film^5^, etc. The real contact area became one of the key factors, as it bears the tangential stress^6^. As a great advance in understanding of friction, Bowden and Tabor^7^ argued that the tangential force required to slide each contact junction is proportional to the junction area by a critical shear strength, which leads to *the linearity between friction force and the real contact area*. This concept can explain the origin of the ancient phenomenological friction law if the linearity between real contact area and normal load is fulfilled.

After the work of Bowden and Tabor, study on the contact of rough surfaces has experienced a shriving development for determining the relation between real contact area and normal load. There are multi-asperities models^8–10^, fractal models^11–13^, Persson theory^14,15^, etc. Interestingly, almost all these theories showed approximately linear load-area relationships within a certain contact fraction^16^. Such linearity was also verified through finite element analyses^17–19^ and atomic simulations^20,21^.

Besides, huge efforts have been devoted into experimental measurements of the real contact area^22^. Indirect methods such as measuring the thermal resistance^23^, electric resistance^24^ and ultrasonic reflection^25^ were adopted to evaluate the contact fraction. To obtain a rather detailed picture of contact spots, techniques as X-ray computed tomography^26^, optical interference^27–29^, fluorescent molecule probes^30^, and frustrated total internal reflection^31–33^ were used for recording the real contact area. Likewise, almost all experiments^26,28,31–33^ reported the linear dependence of real contact area on normal load.

In view of the linear load-area relationship in the continuous loading process, it seems settled that linearity exists between any two of friction force, real contact area, and normal load. Nonetheless, such linearity of load-area relation might break down for some circumstances. For the contact between viscoelastic rubber sphere and glass, a clear hysteresis was found in the load-area curves during the loading–unloading circle^28^. A similar hysteresis was observed for the contact of rough copper sample against smooth glass on account of the plastic deformation^33^. During the unloading process, the real contact area decreases monotonously but nonlinearly with respect to normal load, which may provide a scenario to test whether friction force exclusively depends linearly on contact area or normal load.

In this study, the friction tests are conducted between copper samples and a glass disc. The frustrated total internal reflection method is adopted to measure the real contact area under various external normal load and friction force. The results verify the linear dependence of friction force on both normal load and real contact area during the normal loading stage. Nonetheless, for the unloading stage, such linearity still holds between friction force and normal load, whereas a nonlinear relation between friction force and contact area is observed.

## Experiment

### Sample preparation

**Figure 1:**
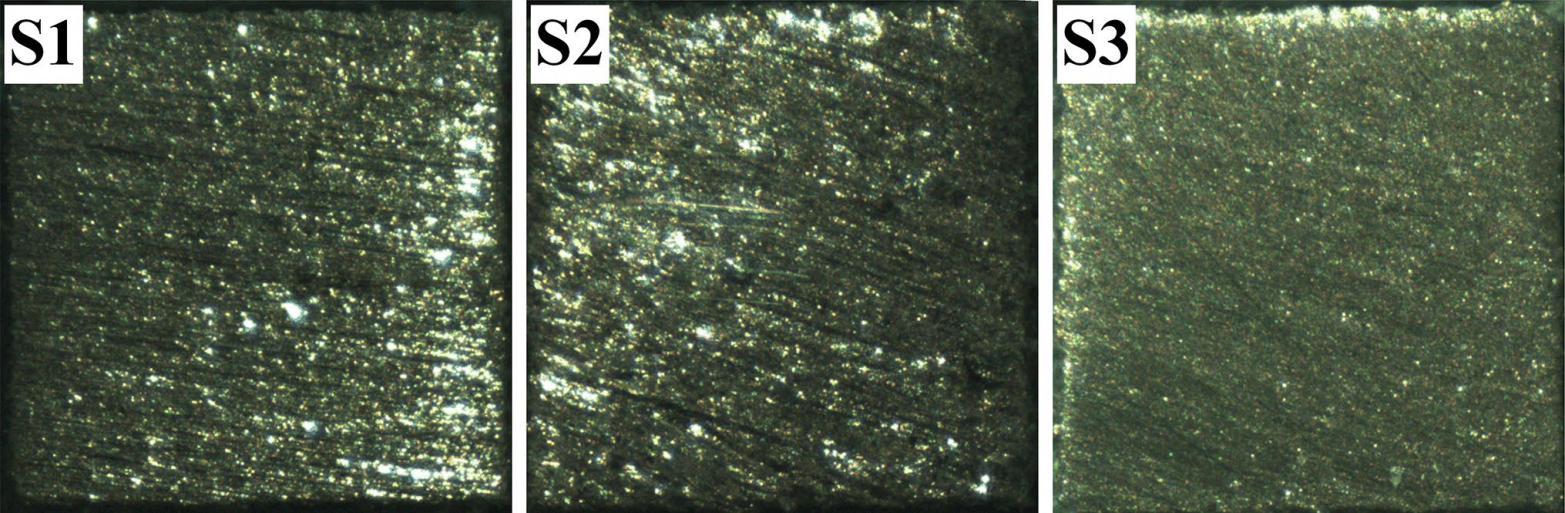
Surfaces of test samples S1, S2, and S3.

Three cubic samples made of copper were prepared for the contact and friction tests. As shown in Fig. 1, the surfaces to be tested were first rubbed with sandpaper to obtain rough topographies. Such surface topographies are quite common for friction pairs. The nominal contact region of each sample surface is of 3 × 3 mm^2^. Prior to the contact-friction tests, standard tensile test was carried out, by which the Young’s modulus *E*_copper_, the Poisson’s ratio *ν*_copper_, and the yield stress *σ*_*y*_ of copper were measured as 125.4 GPa, 0.34, and 225.3 MPa, respectively.

### Experimental apparatus

**Figure 2:**
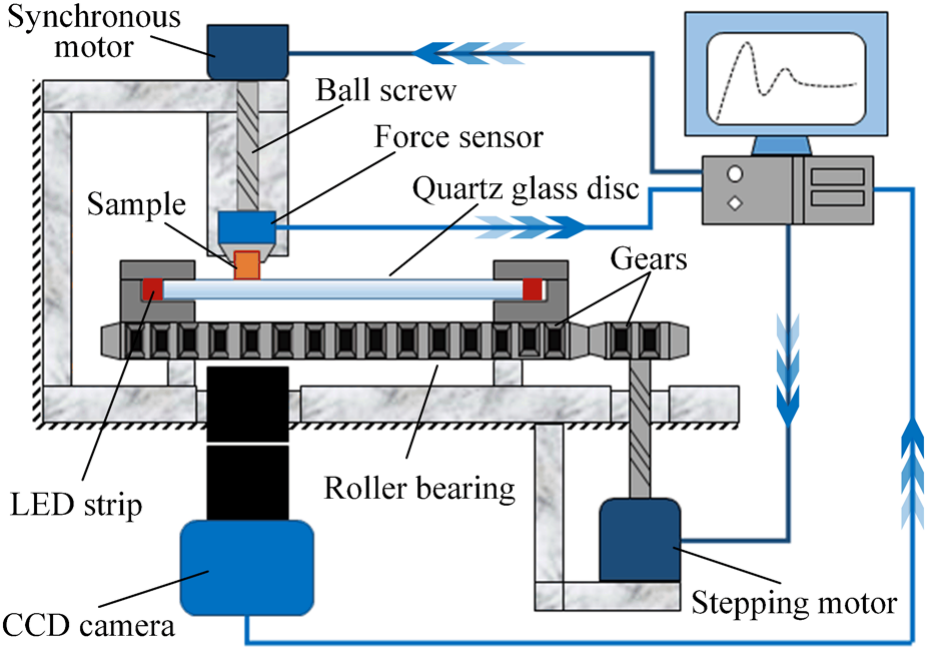
Schematic diagram of the experimental apparatus (generated by Microsoft PowerPoint & Visio 2016 MSO(16.0.4266.1001) 64 bits, http://www.microsoft.com).

As schematically shown in Fig. 2, a new apparatus was designed and built to measure friction force, real contact area and normal load simultaneously. The apparatus consists of three basic parts: the loading system, the illuminating system, and the camera shooting system.

The main module of the loading system is a roller bearing with its inner-ring screwed on a workbench and outer-ring installed with a quartz glass disc. The quartz glass disc is 200 mm in diameter and 15 mm in thickness. A stepping motor is connected with the outer-ring of the bearing through a pair of gears, and the glass disc can rotate steadily with the torque transmitted. The test sample is fixed on a sample holder, which is motivated by a ball screw and a synchronous motor. During contact, the sample will be compressed against the upper surface of the quartz glass at about 40 mm offset from the disc center. When the stepping motor is triggered, the sample will be rubbed by the glass disc, and both shear force and normal load are recorded by a three-dimensional force sensor connected with the sample holder. The loading rate of the sample holder is 0.5 mm/min, and the rotating speed of the glass disk is 0.02 rpm. Therefore, when sliding happens, the relative linear velocity between the sample and the glass disc can be estimated as 0.042 mm/s. The data acquisition rate of the force sensor is set as 30 Hz.

The illuminating system is constructed based on the frustrated total internal reflection technique. A monochromatic violet light-emitting diode strip is wrapping around the glass disc circumferentially. All lights incident into the glass disc from the flank. The light strip and the edge of the glass disc are together covered with a blackout fabric. Lights incident at an angle smaller than the critical total reflection angle are absorbed by the fabric, whereas others remain constrained within the glass disc. The entire apparatus is placed in a dark room to prevent interference from other light sources.

The function of the camera shooting system records the evolution of the real contact area. A charge-coupled device (CCD) camera is installed beneath the glass disc and calibrated to focus on the upper surface of the glass disc. Since the glass disc is much harder than the test samples, its upper surface can be considered as a rigid flat plane, which is easy to focus on during compressing and rubbing. When test sample is compressed against the glass surface, the total internal reflection condition will be broken at the area of intimate contact, and light will be scattered by the sample surface and subsequently captured by the CCD camera^32,33^. Within the non-contact region, light will be reflected at an angle larger than the critical total reflection angle and still trapped inside the glass disc. Once activated, the CCD camera will record the images of the contact interface consecutively at a constant rate of 30 fps. With a 1× objective lens installed on the camera, each pixel on the image corresponds to a squared region of 4.8 × 4.8 μm^2^ on the contact interface.

By adopting the improved Otsu technique^34^, the original images could be transformed into binary images, with 1 representing for contact spots and 0 for non-contact spots. Hence, the real contact area is obtained as the amount of the contact spots times the area of a single spot. Through triggering both camera and force sensor at the same time, the real contact area, the friction force, and the normal load are measured and recorded synchronously.

### Experimental procedure

**Figure 3:**
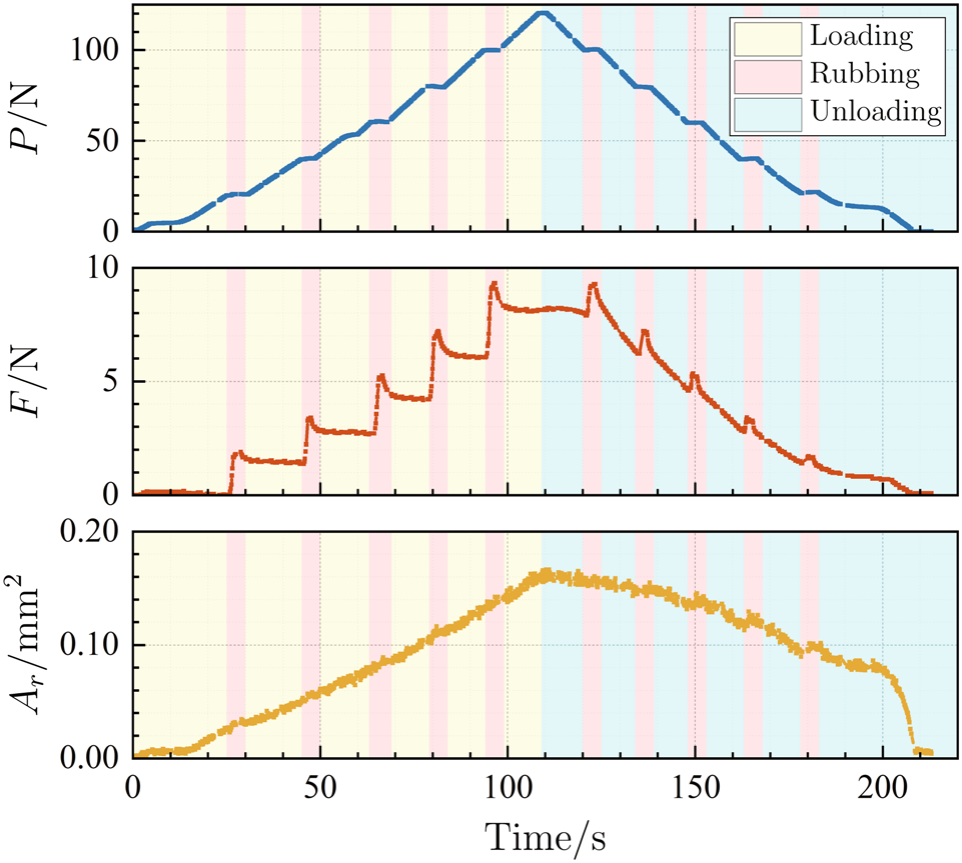
Evolutions of real contact area *A*_r_, normal load *P*, and the friction force *F* versus time for sample S1.

Full normal loading–unloading tests were performed on samples S1, S2, and S3. The normal load *P*, the friction force *F* and the real contact area *A*_*r*_ were continuously measured and recorded in the whole test. As an example, the loading history of sample S1 is shown in Fig. 3. In the loading stages, samples were driven by the synchronous motor and brought into contact against the glass disc, with the normal load *P* gradually increasing from 0 to 120 N. In order to achieve a low loading rate, a coupling and a reduction box were set between the synchronous motor and the ball screw. Possible gaps between these components might cause the normal load stop increasing for a short interval, which resulted in small plateaus in normal load (i.e. around P = 5 N and 53 N) but would not affect the continuity of the loading procedures as well as the correctness of the data acquisition. Then the synchronous motor was reversed, whereupon the normal load *P* decreased till vanishing. During the loading–unloading circle for each sample, a series of rubbing processes were carried out through rotating the glass disc at specific normal loads of 20 N, 40 N, 60 N, 80 N, and 100 N.

The first rubbing process is conducted at about 26 s. Before that time, the friction force keeps almost zero, and the real contact area is very small since the normal load is quite low. It can be seen that the rubbing process consists of two stages. Firstly, the friction force endures a sharp increment till a maximum is reached, commonly known as the maximum static friction force *F*_*s*_. Once the peak value is reached, the friction force rapidly falls, converging to a stable value, recognized as the kinetic friction force *F*_*k*_. In each rubbing process, there is a slight increment of contact area *A*_*r*_, which has also been found in Ref.^35^. To avoid the significant increase of contact area incurred by long time of friction, the rotation of glass disc is immediately halted once the kinetic friction force is detected. In this case, each rubbing process endures about 5 s, and the glass disc will rotate 0.6°. For convenient comparison, *P*, *F*_*s*_ and *F*_*k*_ are normalized by E*A_0_, and *A*_*r*_ normalized by *A*_0_, where E* = *E*_copper_/(1 − *ν*_copper_^2^) is the composite elastic modulus and *A*_0_ represents the nominal contact area.

## Results and discussion

**Figure 4:**
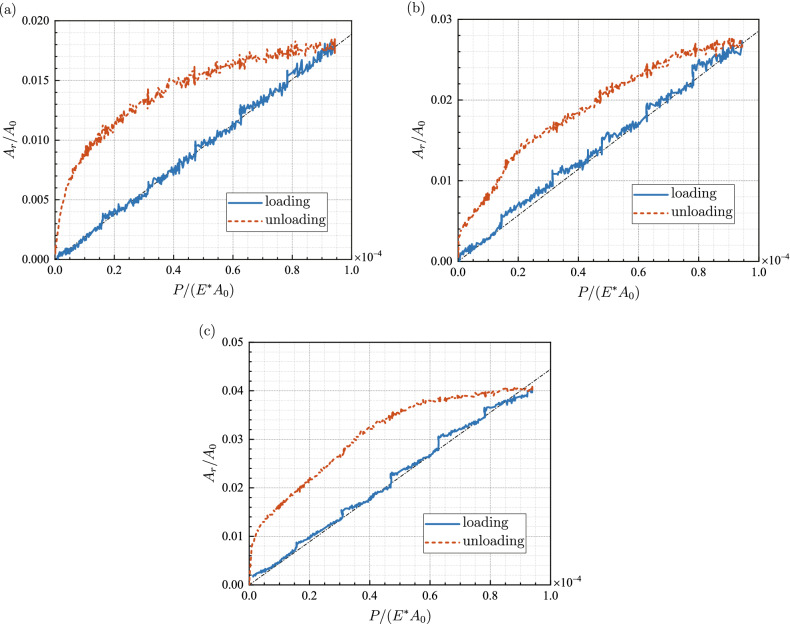
Evolution of the contact fraction *A*_*r*_/*A*_0_ with respect to the dimensionless normal load *P*/(E*A_0_): (**a**) sample S1, (**b**) sample S2, (**c**) sample S3.

**Figure 5:**
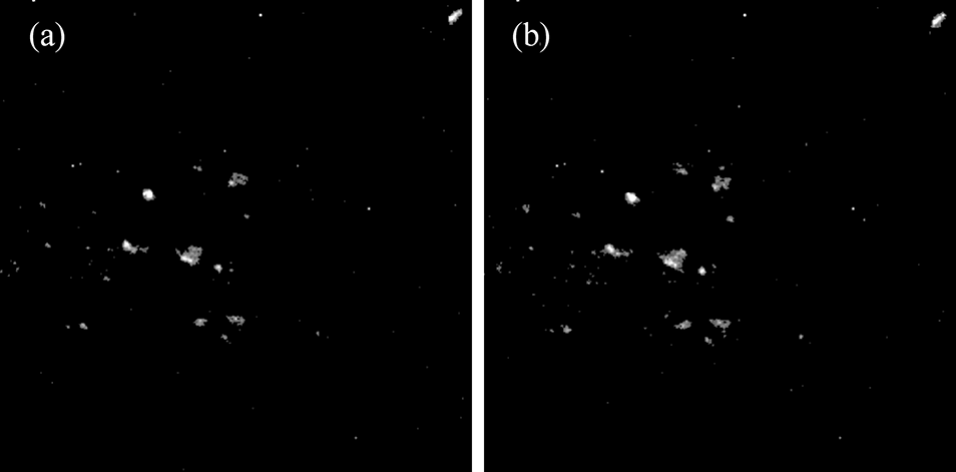
The real contact areas of sample S1 at the: (**a**) initiation of the first rubbing, (**b**) end of the first rubbing.

Figure 4 displays the evolution of the contact fraction *A*_*r*_/*A*_0_ with respect to the dimensionless normal load *P*/(E*A_0_). There are five small jumps in each curve at the loads conducting rubbing processes, where the real contact area endures a small increment owing to friction. For sample S1 under the load *P* = 20 N, the contact regions at the initiation and the end of the rubbing are shown in Fig. 5a,b, respectively. After rubbing, some contact spots have grown larger, along with new ones’ coming into being. Basically, *A*_*r*_/*A*_0_ increases approximately linearly with *P*/(E*A_0_) in the loading stages, and the dimensionless mean pressures *P*/(E*A_*r*_) for samples S1, S2 and S3 are best fitted with 0.0053, 0.0035 and 0.0023, respectively. Correspondingly, the coefficients of determination *R*^2^ take 0.9965, 0.9925 and 0.9945, which demonstrates the efficiency of such linear fittings. However, as the plastic deformation accumulated in the loading stage cannot be recovered, the real contact area decreases monotonously during unloading but nonlinearly till vanishing.

**Figure 6:**
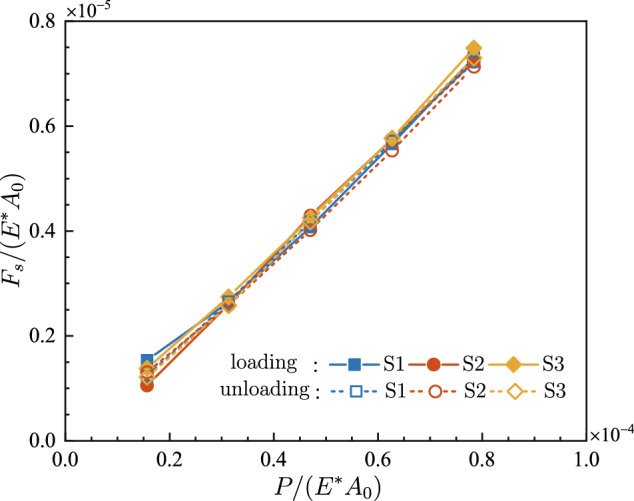
The dimensionless maximum static friction force *F*_*s*_/(E*A_0_) at various dimensionless normal loads *P*/(E*A_0_).

**Figure 7:**
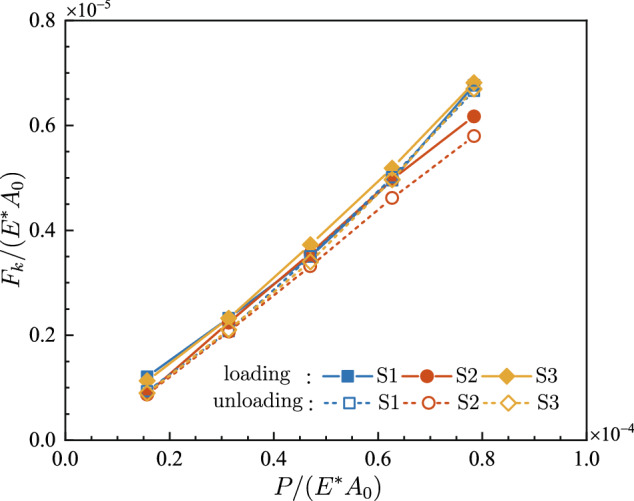
The dimensionless kinetic friction force *F*_*k*_/(E*A_0_) at various dimensionless normal loads *P*/(E*A_0_).

For various normal loads, the dimensionless maximum static friction force *F*_*s*_/(E*A_0_) is presented in Fig. 6. *F*_*s*_/(E*A_0_) is clearly enlarged as the normal load increases. For any specific normal load during both loading and unloading, *F*_*s*_/(E*A_0_) hardly varies among different samples. It appears that both the loading history and rough topography of the samples have little effect on the static friction. Figure 7 displays the dimensionless kinetic friction force *F*_*k*_/(E*A_0_) with respect to the dimensionless normal load *P*/(E*A_0_). The kinetic friction force *F*_*k*_ exhibits a similar dependence on normal load as the static friction force *F*_*s*_ does, except that *F*_*k*_ is a little bit smaller than *F*_*s*_ under the same pressure.

**Figure 8:**
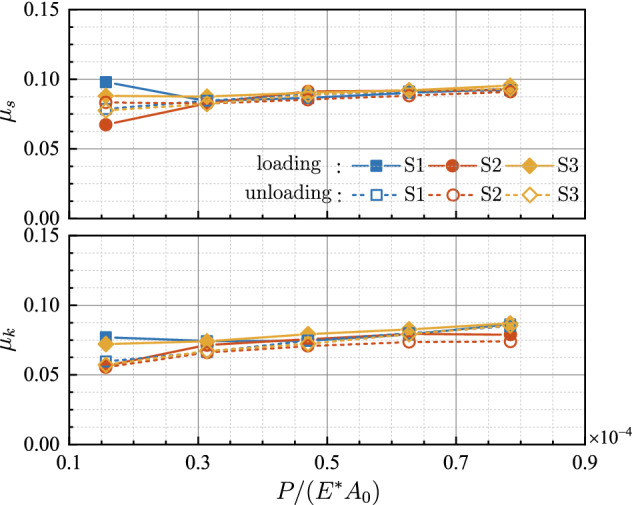
Evolution of the friction coefficient with respect to the dimensionless normal load.

For a clearer insight into the relation between the friction force and the external normal load, the static friction coefficient *μ*_*s*_ and the kinetic friction coefficient *μ*_*k*_ are calculated, as shown in Fig. 8. It is seen that, in both loading and unloading process, *μ*_*s*_ and *μ*_*k*_ keep almost constant, which proves the proportionality between friction force and normal load. For both loading and unloading, the coefficients of determination *R*^2^ for the linear fitting of *F*-*P* curves are presented in Table 1. For each curve, the resultant *R*^2^ exceeds 0.97, showing the reliability of linear relationships.

**Table 1:** The coefficients of determination *R*^2^ after the linear fitting.

Curve	S1	S2	S3
*F*_*s*_-*P*: loading	0.9940	0.9911	0.9960
*F*_*k*_-*P*: loading	0.9826	0.9901	0.9886
*F*_*s*_-*P*: unloading	0.9959	0.9953	0.9931
*F*_*k*_-*P*: unloading	0.9757	0.9911	0.9713
*F*_*s*_-*A*_*r*_: loading	0.9933	0.9804	0.9815
*F*_*k*_-*A*_*r*_: loading	0.9722	0.9808	0.9689
*F*_*s*_-*A*_*r*_: unloading	0.5747	0.7538	0.6780
*F*_*k*_-*A*_*r*_: unloading	0.4940	0.7344	0.6512

**Figure 9:**
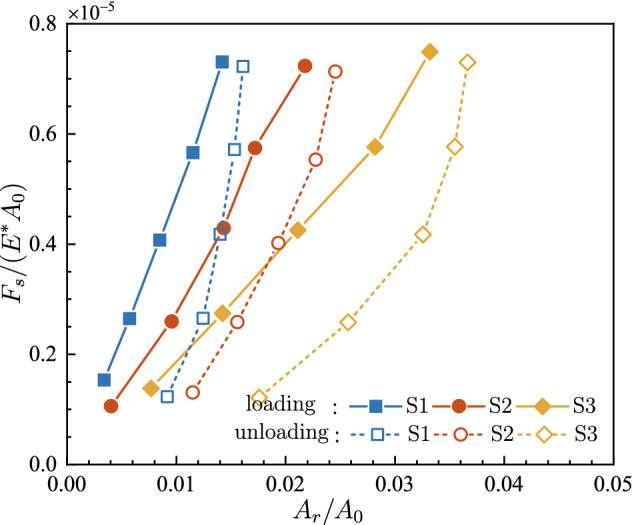
The dimensionless maximum static friction force *F*_*s*_/(E*A_0_) under various contact fractions *A*_*r*_/*A*_0_.

**Figure 10:**
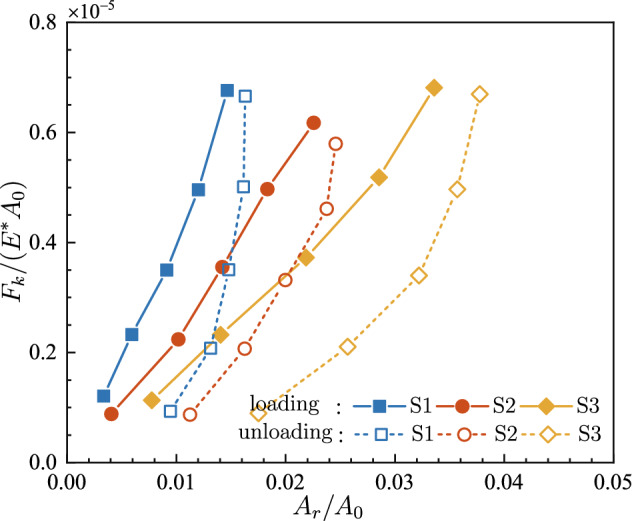
The dimensionless kinetic friction force *F*_*k*_/(E*A_0_) under various contact fractions *A*_*r*_/*A*_0_.

The evolutions of *F*_*s*_/(E*A_0_) and *F*_*k*_/(E*A_0_) versus the contact fraction *A*_*r*_/*A*_0_ are shown in Figs. 9 and 10, respectively. Both friction forces vary in a quite similar pattern with the evolution of the contact fraction. At five specific loads in the loading stage, the frictional force is basically proportional to the contact area. Such linearity was also observed at the onset of friction between PDMS and glass plate^36^. Nevertheless, under the same normal load, thus almost the same frictional force, it is observed that the contact area in the unloading stage is much larger than that in the loading stage.

**Figure 11:**
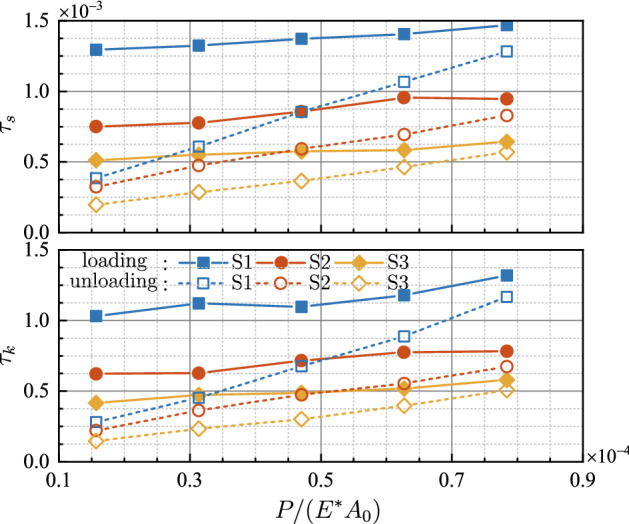
Evolution of the dimensionless mean tangential stress with respect to the dimensionless normal load.

For both static friction and kinetic friction, the dimensionless mean tangential stresses *F*_*s*_/(*GA*_*r*_) and *F*_*k*_/(*GA*_*r*_) are calculated and denoted as *τ*_*s*_ and *τ*_*k*_, respectively, with *G* = *E*/[2(1 + *ν*)] being the shear modulus. Figure 11 displays *τ*_*s*_ and *τ*_*k*_ at various normal loads for both loading and unloading. It can be seen that both *τ*_*s*_ and *τ*_*k*_ change slightly during loading, while decrease monotonously in the unloading stages. The slopes of the dimensionless mean tangential stress with respect to the dimensionless normal load of loading stages are much smaller than those of unloading portions. It suggests that the friction force is approximately proportional to the real contact area at loading stages. The coefficients of determination *R*^2^ for the linear fitting of *F*-*A*_*r*_ curves also lead to the same conclusions. However, *R*^2^ drops less than 0.8 and even down to 0.5 for unloading cases, which proves the breakdown of linearity between friction force and real contact area.

It is also noticed that *τ*_*s*_ and *τ*_*k*_ vary significantly among different samples, which indicates that even for loading the proportional coefficient between friction force and the real contact area is strongly dependent on the rough topographies. A probable explanation is that the variation of the rough topographies results in the variation of the mean pressure on the real contact area, and the mean tangential stress is proportional to the mean pressure by the friction coefficient. Either way, linear dependency does not always hold between friction force and the real contact area, given the fact that the same friction force corresponds to two different values of real contact area during loading and unloading.

For contact between metals and glass, the hysteresis in the load-area curves during the loading–unloading circle comes from plastic deformation^33^. Adhesion is another important factor in analyzing contact surfaces^37^, and may enlarge the real contact area for rough contact^38^. Therefore, the influence of adhesion on the friction-area relation is worth considering. The present work is merely an experimental phenomenological study, the micro-mechanism underlying the observed results is still far from fully understanding and needs further exploring.

## Conclusions

By adopting the frustrated total internal reflection method, an apparatus has been built to measure the friction force, the real contact area, and the external normal load in-situ and real-time. Loading–unloading tests are conducted on various copper samples. At several specified normal loads, samples are rubbed for a short period of time in both loading and unloading stages. The experiment results suggest that the friction force is roughly proportional to both the normal load and the real contact area during loading. Such linearity still holds between friction force and normal load during unloading, while fails for the relation between friction force and real contact area. These results illuminate that the linear assumption of friction force on contact area does not always hold.
